# Development and validation of a reliable DNA copy-number-based machine learning algorithm (*CopyClust*) for breast cancer integrative cluster classification

**DOI:** 10.1038/s41598-024-62724-6

**Published:** 2024-05-24

**Authors:** Cameron C. Young, Katherine Eason, Raquel Manzano Garcia, Richard Moulange, Sach Mukherjee, Suet-Feung Chin, Carlos Caldas, Oscar M. Rueda

**Affiliations:** 1 Cancer Research UK Cambridge Institute and Department of Oncology, Li Ka Shing Centre, University of Cambridge, Cambridge, UK; 2 Harvard Medical School, Boston, MA USA; 3 MRC Biostatistics Unit, University of Cambridge, East Forvie Building, Forvie Site, Robinson Way, Cambridge Biomedical Campus, Cambridge, CB2 0SR UK; 4 Deutsches Zentrum für Neurodegenerative Erkrankungen (DZNE), Bonn, Germany; 5 University of Bonn, Bonn, Germany

**Keywords:** Breast cancer, Machine learning

## Abstract

The Integrative Cluster subtypes (IntClusts) provide a framework for the classification of breast cancer tumors into 10 distinct groups based on copy number and gene expression, each with unique biological drivers of disease and clinical prognoses. Gene expression data is often lacking, and accurate classification of samples into IntClusts with copy number data alone is essential. Current classification methods achieve low accuracy when gene expression data are absent, warranting the development of new approaches to IntClust classification. Copy number data from 1980 breast cancer samples from METABRIC was used to train multiclass XGBoost machine learning algorithms (CopyClust). A piecewise constant fit was applied to the average copy number profile of each IntClust and unique breakpoints across the 10 profiles were identified and converted into ~ 500 genomic regions used as features for CopyClust. These models consisted of two approaches: a 10-class model with the final IntClust label predicted by a single multiclass model and a 6-class model with binary reclassification in which four pairs of IntClusts were combined for initial multiclass classification. Performance was validated on the TCGA dataset, with copy number data generated from both SNP arrays and WES platforms. CopyClust achieved 81% and 79% overall accuracy with the TCGA SNP and WES datasets, respectively, a nine-percentage point or greater improvement in overall IntClust subtype classification accuracy. CopyClust achieves a significant improvement over current methods in classification accuracy of IntClust subtypes for samples without available gene expression data and is an easily implementable algorithm for IntClust classification of breast cancer samples with copy number data.

## Introduction

Heterogeneity is one of the main characteristics of breast cancer, and this is present both in the biology of the disease and the clinical management of patients. Starting from the basic estrogen receptor (ER) and human epidermal growth factor 2 (Her2) tumor stratification, which led to targeted treatment for breast cancer (hormone therapy and anti-Her2 therapy), efforts have moved to molecular stratification of tumors. In a landmark study^1^ five intrinsic subtypes were defined based in patterns of RNA expression, named Basal, Her2, Luminal A, Luminal B and Normal-like. A classifier system called PAM50 was later derived to assign one of these groups to any tumor based on its expression profile^2^. Other taxonomies were later proposed, for example to subdivide ER- tumors,^3^ to add a new group, claudin-low to the intrinsic subtypes^4^ or to refine ER + tumors^5^ using multi-omic data.

The rationale employed to select the features that will be used to identify subgroups will guide the taxonomy features. With the aim of identifying different cancer driver genes, we selected 1,000 genes that showed a paired copy number aberration and differential expression on the METABRIC cohort^6^. Using an integrative clustering approach^7^ we identified 10 unique breast cancer tumor subtypes (Integrative Clusters [IntClusts]) each with characteristic genomic and transcriptomic architecture and genomic driver events^6^. In subsequent studies, we fully characterized these subtypes in terms of miRNAs activity^8^, somatic mutations^9^, methylation profiles^10^, and relapse patterns^11^. A classifier that uses a set of copy number and expression profiles was developed and validated in several cohorts^12^ and made available as an R package (*iC10*)^13^.

These studies show that the IntClusts are distinct biological entities with different molecular features and clinical outcomes, possibly benefiting from individualized treatments, and provide a framework for personalized breast cancer therapeutic strategies with extensive clinical utility^14,15^. For clinical application, new samples, which are initially unlabelled, must be assigned to a class. However, the current recommended approach to classify unlabelled tumor samples into IntClusts rely on using a combined copy number and gene expression focused approach^12^, limiting classification accuracy among cancer samples without transcriptomic data. Although the R package *iC10* allows classification with only copy number data, performance is lower compared to using expression data^12^. With the increase in cancer genomics consortia and widespread availability of public data without gene expression profiling, the need for a novel method to subtype tumors from independent cohorts based on copy number data alone is warranted. Here, we present the development and validation of a reliable, flexible, platform-independent copy number-driven machine learning algorithm (*CopyClust*) for IntClust classification as an open-source R package.

## Methods

The 1980 breast cancer samples from METABRIC (internal validation) and the 1075 samples from TCGA (external validation) with available copy number and gene expression data were used to train and validate multiclass hyperparameter-optimized XGBoost^16^ machine learning algorithms. For METABRIC, IntClust label was assigned from the original manuscript^6^, while for TCGA, label was assigned via the *iC10* classifier^12,13^ using copy number and gene expression data. To reduce noise, a piecewise constant fit (PCF) was applied to the copy number profiles of the METABRIC samples in each IntClust and unique breakpoints across the 10 profiles were identified and converted into 478 genomic regions. The mean copy number in each region was calculated, and these were used as features for XGBoost models. METABRIC samples were split into a training cohort (80%) and validation cohort (20%). Sixteen intra-IntClust outliers were identified via local outlier factor (LOF) and removed from the training cohort prior to model training.

XGBoost modeling was performed using the framework provided by the *xgboost* R package (v1.7.3.1)^17^. To perform hyperparameter optimization, the XGBoost machine learning models were subjected to stratified fivefold cross-validation in which 20% of the training dataset was excluded from each fold and used as validation. Each sample only appeared in a single fold and each fold contained an equal distribution of IntClusts. These folds were iterated through treating each one as the validation set in each iteration, with the remaining four folds combined as the training set. The performance of the hyperparameters was assessed using the independent, unseen, held-out validation fold. These iterations were repeated for various sets of hyperparameters selected via random search optimization, which performs better than grid search or manual search optimization^18^. The hyperparameters that resulted in the lowest mean objective value (log-loss score for multiclass models and root square mean error for binary models) were selected for use in the final models and these models were trained using the entire training dataset.

**Figure 1:**
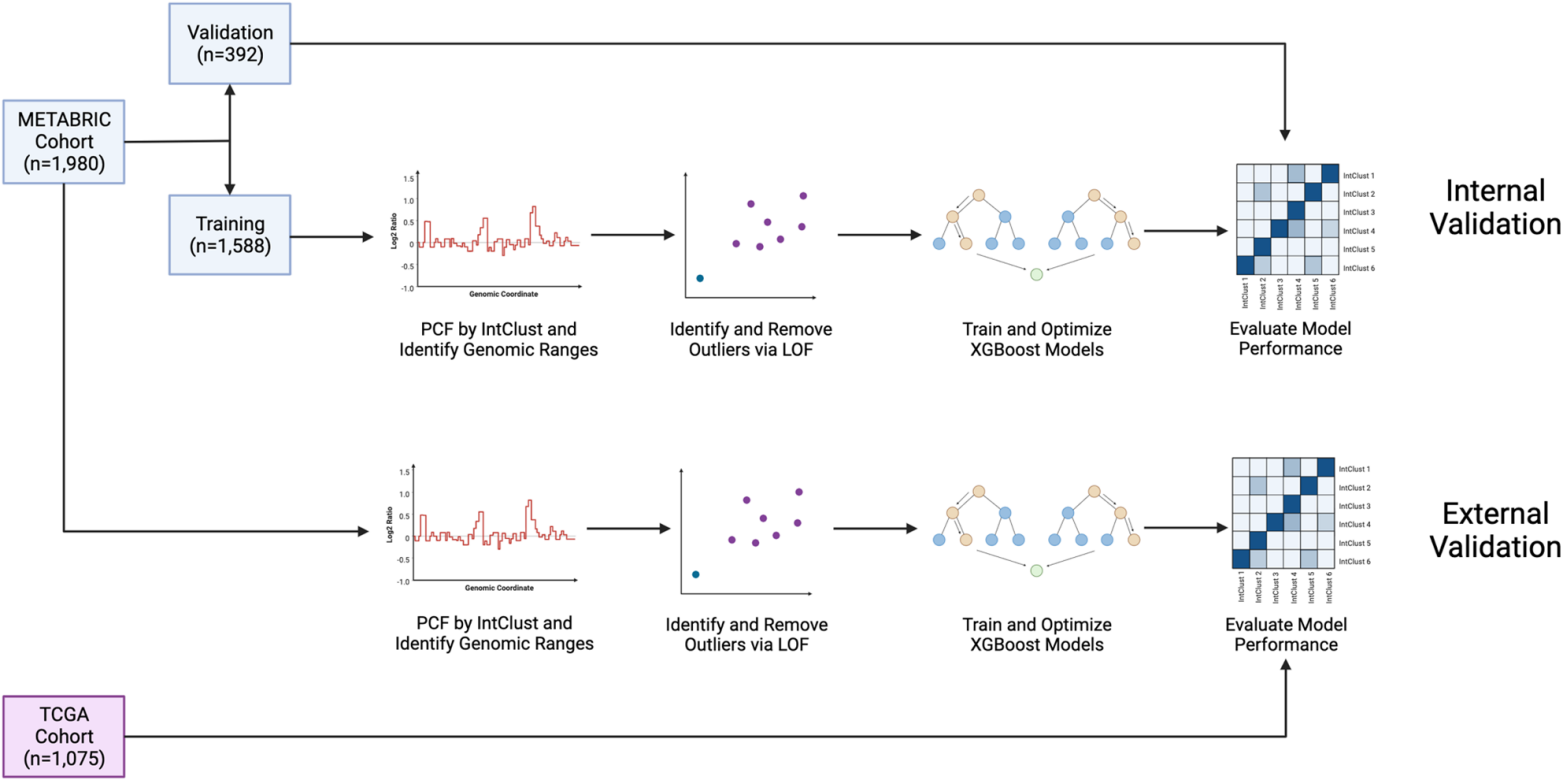
Workflow of algorithm development for internal and external validation.

Two model approaches were implemented: a 10-class model with the final IntClust label predicted by a single multiclass model and a 6-class model with binary reclassification in which four pairs of IntClusts were combined for initial multiclass classification, then assigned an IntClust label based on the prediction of a second binary classifier trained on that pair. As a reduction in the number of classes and combination of similar classes in a multiclass model has been shown to increase performance^19^, and multiple binary models tend to perform better than multiclass models^20^, pairs of IntClusts with similar mean copy number profiles were combined and binary models were trained and optimized using only samples from the training cohort that belonged to the two IntClust groups of interest. Multiclass models with different numbers of combined IntClust pairs were assessed, and a 6-class model with four pairs of combined IntClusts was selected due to superior performance. These pairs consisted of: IntClusts 1 and 5, IntClusts 3 and 8, IntClusts 4 and 7, and IntClusts 9 and 10 and were selected based on their similar copy number profiles (Supplementary Figures S6–S15) and frequent misclassification in the 10-class model (Supplementary Figure S1). The 6-class model with binary reclassification model was selected for final model implementation due to superior performance (Supplementary Table S1). Scaling of feature values was performed prior to model implementation on external cohorts. Model performance was internally validated on 392 (20%) held-out METABRIC samples and externally validated on the TCGA dataset, with copy number data generated from both single nucleotide polymorphism (SNP) arrays and whole exome sequencing (WES) (Fig. 1). More details of the methodology used and results for this study can be found in the Supplemental Methods and Supplemental Tables S1–S7 and Supplemental Figures S1–S19.

## Results and discussion

**Figure 2:**
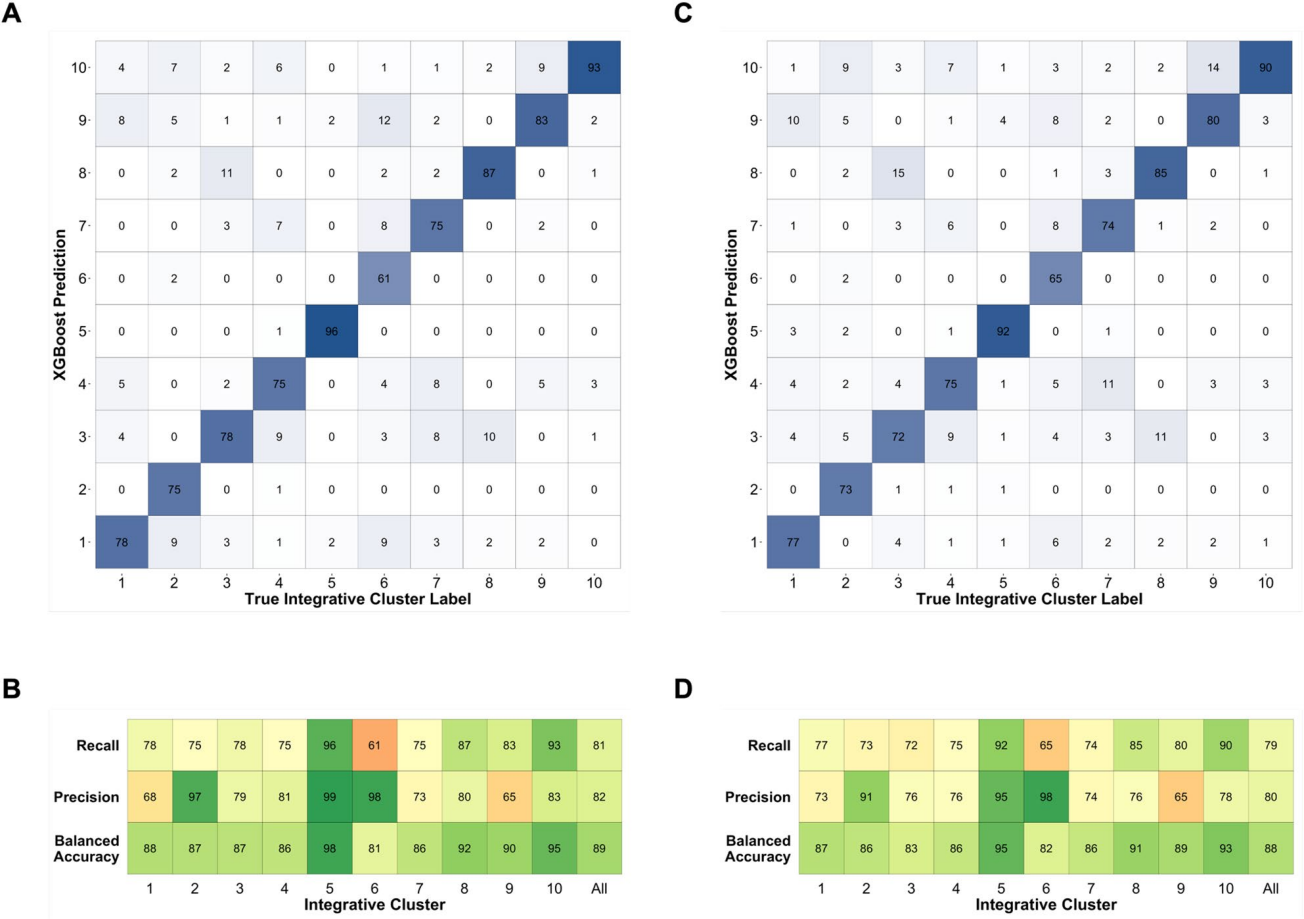
Performance of *CopyClust* on IntClust Label Assignment of TCGA SNP and WES Cohorts. (**A**) Confusion matrix of true IntClust label of TCGA SNP cohort (x-axis) and *CopyClust* prediction (y-axis). Values represent percentage of true IntClust label predicted to be in each class by *CopyClust*. The diagonal represents the percent of samples correctly predicted as a particular IntClust and is equivalent to recall. (**B**) Model performance metrics, where recall = percentage of correctly classified samples per IntClust; precision = percent of correctly classified samples amongst samples predicted as a particular IntClust; and balanced accuracy = mean of recall and specificity. (**C**) Confusion matrix of true IntClust label of TCGA WES cohort (x-axis) and *CopyClust* prediction (y-axis). Values represent percentage of true IntClust label predicted to be in each class by *CopyClust*. The diagonal represents the percent of samples correctly predicted as a particular IntClust and is equivalent to recall. (**D**) Model performance metrics as in B. Overall performance metrics above the “All” column are reported as micro-averages across all IntClusts.

When compared to other hyperparameter-optimized classifier algorithms including Random Forest, Support Vector Machine, LightGBM, and Prediction Analysis of Microarrays, XGBoost performed best in terms of overall recall and Matthews Correlation Coefficient (MCC) (Table 1) and was selected as the approach for *CopyClust*. *CopyClust* achieved high classification performance across both the TCGA SNP and WES datasets (Table 1 and Fig. 2). The classifier produced an overall recall of 81%, precision of 82%, and balanced accuracy of 89% when applied to the TCGA SNP dataset with an F1 Score of 0.811 and MCC of 0.787. Applied to the TCGA WES dataset, *CopyClust* produced an overall recall of 79%, precision of 80%, balanced accuracy of 88%, F1 Score of 0.786, and MCC of 0.759 (Table 2). Across both datasets, IntClust 3 and IntClust 8 were the most misclassified pair of IntClusts, likely due to their similar copy number profiles. IntClust 6 experienced the lowest individual recall, which could be due to differences in the distribution of IntClusts between cohorts (Supplementary Figure S2), leading to model miscalibration^21^.

**Table 1:** Overall recall of different classifier approaches.

Model approach^a^	Overall recall (95% confidence interval)^b^	Matthews correlation coefficient
Random forest	68.4% (65.5%, 71.1%)	0.651
Support vector machine	68.6% (65.7%, 71.3%)	0.645
LightGBM	71.4% (68.5%, 74.0%)	0.677
Prediction analysis of microarrays	66.8% (63.9%, 69.6%)	0.628
XGBoost – TCGA SNP Cohort	81.1% (78.7%, 83.4%)	0.787
XGBoost – TCGA WES Cohort	78.6% (76.0%, 81.0%)	0.759

^a^Models were applied to TCGA SNP cohort unless otherwise specified.

^b^Overall recall is reported as micro-average across all IntClusts.

**Table 2:** Performance metrics of *CopyClust* model on TCGA SNP and WES cohorts.

Sample cohort	Recall	Precision	Balanced accuracy	Specificity	F1 Score	Matthews correlation coefficient
TCGA SNP	0.811	0.823	0.893	0.975	0.811	0.787
TCGA WES	0.786	0.796	0.879	0.971	0.786	0.759

Metrics reported as micro-average across all IntClusts.

Compared to the current gold standard copy number-only *iC10* classifier, *CopyClust* achieved a nine-percentage point greater overall recall when applied to METABRIC (82% vs. 73%) and a 22% and 20% greater overall recall when applied to the TCGA SNP and WES datasets, respectively (81% and 79% vs. 59%) (Supplemental Table 1). This increase in the performance of *CopyClust* compared to the *iC10* classifier can likely be attributed to the dominance of gene expression features in the selected probes of the *iC10* classifier^12^. Features in the *iC10* classifier were taken from the original IntClust manuscript^6^, and only 38 out of 714 (5.3%) of the probes used are gene copy number; therefore, the bulk of the features are gene expression values. The *iC10* classifier is trained using the prediction analysis of microarrays shrunken centroids approach, which was developed for gene expression analysis^22^. Rather than using a small subset of copy number probes, *CopyClust* was trained using features comprising the entire length of the genome. Many IntClusts have key features of their copy number profiles that are characteristic for a given IntClust^15^ (e.g. IntClust 5 [chromosome 17q12 amplification] and IntClust 6 [chromosome 8p12 amplification]). The copy number probes used by the *iC10* classifier do cover some of these key regions, but they do not cover the characteristics of the entire copy number profile, indicating the superiority of *CopyClust* in the absence of gene expression profiling.

Intricacies of the specific datasets used to train and validate *CopyClust* somewhat limit its generalizability. METABRIC did not set a minimum tumor cellularity^6^, while TCGA set a minimum of 60%^23^. The TCGA cohort may be composed of tumors with a greater average percentage of neoplastic cells; this difference may also account for the stronger signal observed in the TCGA copy number profiles relative to the METABRIC copy number profiles (Supplementary Figure S18), which necessitated feature scaling before model training. Additionally, the need to apply feature scaling across samples limits performance when there are only a single or few samples to classify. Manual curation of genomic ranges developed from PCF was only performed to ensure that ranges did not span multiple chromosomes but ranges still cover regions of telomeres and centromeres. Finally, *CopyClust* was only trained using a single cohort and validated externally on a single cohort, therefore, replication on additional datasets may further improve performance.

*CopyClust* provides an accurate and easily implementable framework for IntClust classification using copy number data and achieves a nine-percentage point or greater improvement in overall classification recall compared to the current gold standard approach. Furthermore, *CopyClust* can flexibly handle missing features, is agnostic to differences in genomic profiling platforms, and is easily implementable in an open-source environment, allowing for seamless application to external genomic datasets. The *CopyClust* R package is currently available for download on GitHub (https://github.com/camyoung54/CopyClust).

### Supplementary Information

Supplementary Tables.

Supplementary Information.

## Data Availability

The datasets generated and/or analyzed during the current study are available in cBioPortal (METABRIC: https://www.cbioportal.org/study/summary?id=brca_metabric; TCGA: https://www.cbioportal.org/study/summary?id=brca_tcga).

## Code availability

The underlying code for this study is available in GitHub and can be accessed via this link https://github.com/camyoung54/CopyClust.
